# Supplementary material for: Data-driven models for atmospheric air temperature forecasting at a continental climate region
Source: PLoS One. 2022 Nov 3;17(11):e0277079. doi: 10.1371/journal.pone.0277079 (PMC9632800; doi:10.1371/journal.pone.0277079)
Supplement: Attachment — Submitted filename: Response to Reviewers.docx [file pone.0277079.s001.docx]

Dear Editor,

Thank you for giving us the opportunity to submit a revised draft of our manuscript titled
"Data-Driven Models for Atmospheric Air Temperature Forecasting at a Continental Climate Region" to *PLOS One journal.* We appreciate the time and effort that you and the reviewers have dedicated to providing your valuable feedback on our manuscript. We are grateful to the reviewers for their insightful comments on our paper. We have been able to incorporate changes to reflect the suggestions provided by the reviewers. We have highlighted the changes within the manuscript. Furthermore, the problem related to copyright issues (regarding to figure 1) has also been addressed.

Here is a point-by-point response to the reviewers' comments and concerns.

Comments from Reviewer 1

Reviewer #1: The paper presents an interesting case study on applying four machine learning models to forecast air temperature in Cray station of North Dakota using selected historical temperature as features. The forecasting performance of the four models are compared. There are several aspects that are not adequately addressed by the paper (see the major comments below), making the comparison results less solid.

Major comments:

1. As shown in Figure 6. the temperature data in the paper exhibit strong seasonality. A common practice for seasonal time-series data is to decompose the data into a seasonal component and a deseasonalized component, and then use ML models to predict for the latter. This usually improves forecast performance since some of the temporal dependence in the data can be captured by the seasonal models. The paper should also compare with the ML models using deseasonalized data.

Reply: Thank you for raising this matter. Decomposing the time series data may improve the model performance. One of the most famous techniques is wavelet transform. In literature, there are lots of scholars used this technique and the prediction accuracy had improved significantly. However, recent studies showed that this approach is not suitable for prediction purposes[1]. Accordingly, we have used valid technique called Holt-Winters approach [2] to decompose the time series data into three components level, trend, and seasonality. The Holt-Winters approach is required to tune the three coefficients (α, ß, and ɣ). We have applied a genetic algorithm for finding the optimal values of these coefficients. However, after the decomposition has been completed, the time series components have been faded to AI models to predict the temperature. Unfortunately, the forecasting accuracy has deteriorated. Please see the results in the table below.

Table: the effects of decomposition on the models’ performances: daily scale.

| Before | | | | | After | | | | |
| --- | --- | --- | --- | --- | --- | --- | --- | --- | --- |
| Model | RMSE | R | U | MAE | Model | RMSE | R | U | MAE |
| GBR | 3.612 | 0.963 | 0.128 | 2.791 | GBR | 4.153 | 0.952 | 0.153 | 3.228 |
| QRT | 3.691 | 0.961 | 0.13 | 2.835 | QRT | 4.363 | 0.952 | 0.160 | 3.344 |
| SVR | 3.592 | 0.964 | 0.127 | 2.745 | SVR | 3.911 | 0.956 | 0.142 | 2.984 |
| RT | 3.67 | 0.962 | 0.13 | 2.831 | RT | 4.361 | 0.948 | 0.161 | 3.375 |
| RF | 1.776 | 0.991 | 0.063 | 1.353 | RF | 4.369 | 0.949 | 0.161 | 3.399 |

2. It is unclear to me how tuning parameters are chosen for each ML model using the "trial- and error- method," and hence the performance comparison in the paper is not entirely convincing (and the results may not be reproducible either). I think the paper should rigorous describe the tuning parameter selection procedure, including but not limited to: What are the hyperparameters being tuned? What are their candidate values? What are the criteria to select the optimum values? What are the selected hyperparameters for the results reported in Section 3?
Reply: Done
3. I think more details and justifications are needed on using autocorrelation function (ACF) and partial autocorrelation function (PACF) to choose the number of lags in the candidate models (Table 3). While it seems that the paper is using the lags that lead to PACF values larger than a certain threshold to determine the features included in the daily models, this does not seem to be the case for the weekly models. Also, the weekly models in general achieve better performance by including more features. Does including lag 8 and beyond further improve the performance?
Reply:

1- Justification with essential equations is included in the manuscript.
2- We accordingly have included lag 8 and trained the AI models however, we found that the accuracy could not be improved significantly. This may be because of the redundant information that has been used. We have provided a discussion regarding this matter.

4. The paper should include the performance of traditional time-series models (e.g., ARIMA) as a benchmark, otherwise it is hard to gauge the performance of the four ML models. There are also many off-the-shelf data-based approaches for univariate time-series forecasting, such as THETA [1], Prophet [2] and TBATS [3]. It would be interesting to see what their performance would be for this data set.
Reply: ARIMA model has been used as a benchmark accordingly.

5. It is unclear how forecast is performed on the test data set. For example, to forecast $T_t$ on 2020-1-1 using M2, do you plug in the actual values of $T_{t-1}$ and $T_{t-3}$, or do you plug in predicted values of them? From a practical perspective, these two approaches correspond to a one-step-ahead forecast and a multiple-step-ahead forecast, respectively. The paper should clarify which of these two types of forecasting is considered.

Reply

- The inputs were only previous values not predicted temperatures.
- ARIMA model was used as a benchmark model as suggested.
- One step ahead was used.
- All above information were included in the manuscript.

6. The paper compares RT and QRT with GBR. I would recommend also comparing their performance with random forest [4] and quantile regression forest [5], which are known to have lower prediction variance than RT and QRT, respectively.

Reply: Random Forest has been used accordingly.
7. In Section 4, the paper summarizes the forecast errors by scatter plots, histograms, and boxplots, which seem redundant to me as they all convey similar information on the model performance. In addition, it would be interesting to see a plot of residuals and/or errors against the time, from which one can examine the temporal patterns of the residuals/errors. A strong temporal dependence in the residuals may suggest that the model is underfitting the dependence among the data and more complex models may be needed.

Reply: residual plots for each model have been established as suggested.

Minor comments:
1. What is the same size of the training and test data sets in each model?

Reply: all the details have been included in the manuscript. Please see the model development section.
2. In the second paragraph of Section 2.1, what are the reported summary statistics? The standard deviation for July does not seem correct.

Reply: it is corrected.
3. In Figure 8, are the models for different months trained by the same data set from 2000-2015, or the model for a particular month is trained by the data from the same month (e.g., the model for January is trained by the January data only)? Due to the strong seasonality in the data, the results from the two approaches may be different.

Reply: this figure was created to help us to find a significant difference between the models. Actually, all models were trained using data from 2000 to 2015. we did not train the models for specific months. Eventually, almost all the models provided similar accuracy. However, in the previous version of the paper, we could not use the random forest model. After we use the random forest model in this current version, we found that there is a significant difference between that model (random forest) and the models that have already been applied.

4. In Equation (1) and (3), there should be a transpose sign between w and x. The optimization formulation in Equation (2) and (3) should include the "slack variables."
Reply: Done
5. The mathematical notations should be clearly defined and used consistently. For example, throughout the paper, $\gamma$, $y$ and $T$ are all used to denote the dependent variable. Equations in Section 2.5 are not correctly numbered.
Reply: Done.
6. The writing in Section 3 could be improved. For example, the detailed discussion on model fitting during the training phase seems unnecessary and could be misleading as the main focus of the paper is on out-of-sample forecasting.
Reply: it is improved and motivated as suggested.

**References**

[1]	J. Quilty and J. Adamowski, “Addressing the incorrect usage of wavelet-based hydrological and water resources forecasting models for real-world applications with best practices and a new forecasting framework,” *J. Hydrol.*, 2018, doi: 10.1016/j.jhydrol.2018.05.003.

[2]	L. Liu and L. Wu, “Holt–Winters model with grey generating operator and its application,” *Commun. Stat. - Theory Methods*, vol. 51, no. 11, pp. 3542–3555, Jun. 2022, doi: 10.1080/03610926.2020.1797804.

Comments from Reviewer 2

Reviewer #2: Review of: Data-Driven Models for Atmospheric Air Temperature Forecasting at a Continental Climate Region.

This paper uses multiple machine-learning or deep-learning methods to predict daily and weekly air temperature, given the air temperature of previous days (or weeks). Since machine learning and deep learning became one of the main techniques that could be used in various fields, application of these methods in weather and atmospheric science has been growing. In that sense, I think this paper could be one of the case studies of application of ML and DL methods in weather prediction.
However, there is some concerns regarding the performance of the model. I think the performance of the developed model is not sufficient to claim that the authors are actually making the prediction of air temperature. Additional analysis to increase the model performance is needed for this paper to be considered for publication.

Major comments:
1. Section 2.2 – 2.4
These methodologies are widely used, and it is unnecessary to explain the standard procedures for each method. What is important is to highlight the advantage of each model and variation of each model that the authors made to be more suitable for this study. For example:
1) the authors used RBF kernel for SVR. What is the bandwidth of the kernel? Is there a regularization parameter?

Reply: Thank you very much for your constructive comments. the bandwidth and regularization parameter are explained and defined.
2) DT methods, what is the depths of the trees? What are the number of samples required to split?

Reply: they are included accordingly.
3) Elaborate the pruning method. How is this done?

Reply: Done
4) GBR, what is the learning rate? What is the depth of the model?

Reply: they are included accordingly.
Overall, there are so many hyperparameters that authors selected, but none of these values were given. It will be more informative to give this number than to explain the standard model background. Furthermore, with the values of hyperparameters given, it is essential to perform a sensitivity test on the hyperparameters – authors note that trial and error with 100 iteration was used for this. Was there a big difference in the performance of the model? What are the selected parameters?

Reply: the hyperparameters of all applied models have been defined in the manuscript. We also provide the candidate values of these parameters and explain the criteria of selection of the model through training process. Finally, we provided the hyperparameters of the best model in this study for both scales (daily and weekly).
2. Please elaborate the ACF and PACF (such as equations). Why are these functions used to determine the best lags?

Reply: Done.
3. On the other hand, despite the RT and QRT models showing the best performance during the training phase, they came last during the testing phase since they may overfit the data during the training phase.
Did authors fix the hyperparameters to prevent the overfitting? I think if the authors guess that overfitting happened, they should fix the models.

Reply: we trained models using several hyperparameters for many times and we select the best model. However, in the previous manuscript, there were slight differences between the comparable models. Some researchers confirm that the decision tree models are usually overfitting the data compared to other models [3], [4]. This is the nature of that models so that the scholars developed the random forest and other assembling models to reduce overfitting problems[5] . However, in the current manuscript we used random forest model and the result of than model was much better form others. The random forest models have less variance compared to other models and hence predicting temperature more efficiently.

4. For the daily predictions, MAE of the best model (SVR) is about 2.7degC, and for weekly predictions, MAE is 3.3degC. The problem with these numbers is that we don’t know how good these values are. So, there should be some kind of baseline to compare to see how good these results are. For example, what would be the MAE of daily predictions if we just use the previous day’s temperature as prediction? Or, for example, what if we just use the seasonality of temperature for the prediction?

Reply: we have used ARIMA model as a baseline model accordingly. Moreover, we also use random forest (RF) as a modern model. Interestingly, the RF model provides better predictions than other models. There is a significant reduction in MAE after using RF. Furthermore, the RF model were established to predict the future temperature based on only previous day record.

5. One of the concerns is that 30% of the data at the end of the observation is used as testing dataset. As authors mentioned in the introduction, the climate is changing. So, the physical drivers of temperature as well as mean and trend of temperature and change during the observation. The authors can use 30% of randomly selected data as a testing data or add in “year” term to the model and see how the results change.

Reply: Done.
6. More detailed assessment of the predictions should be made. For example, looking at Fig. 10, the prediction errors are higher in colder temperatures. Naturally this is reasonable since temperature variability is higher in winter. Seasonal analysis of temperature should be included. – Now that I look at Figure 8, I see the seasonal analysis. Please talk about this in the main text, and please provide some insights on increase of error in winter is happening.

Reply: Done.

7. The authors discussed outliers (or extreme temperature events) using the IQR. One of the most important features in forecasting temperature is ability to capture the extreme temperatures. So, rather than using IQR with all datasets, authors should see how the models capture the seasonal extreme temperatures (for example, 2sigma values in June, July August, for heat extreme).

Reply: the models’ performances have been assessed through the hottest months accordingly.
8. Overall, I don’t think the model is doing a good job on forecasting temperature. I took the referenced data (is it Cray or Crary? – I could not find station named Cray). For the 2000-2021 period, I simply compared daily temperature and the daily temperature the day before, and the RMSE was 3.83 degC for the entire period and MAE was 2.91 degC. I mentioned this in the previous comment, but what this means is that the model is not significantly better than simply taking the temperature of day before as its predictions. Furthermore, mean error with my calculation was -0.0015, and STD was 3.83. Comparing this with Fig 11, there is no significant improvement in the model. Attempt to utilize ML to forecast temperature is indeed valuable, but better model performance is needed for this research to be justified.

Reply:

1. The station’s name has been corrected.
2. Two additional models (ARIMA, and random forest models) have been performed as suggested. We found that the random forest model has less variance than other models and thus predicting the temperature very well. Eventually, all the result sections and discussions have been changed and motivated.

   Minor comments:
   It is well known that numerous meteorological and ecological events, human life, and crops in agricultural areas are significantly influenced by climate conditions as well as the environment's physical conditions.
   - “Environment’s physical conditions” are rather vague. Please clarify or give examples.
   Reply: Done.
   Given these climate conditions, the temperature is a critical factor that can change and is one of the most significant meteorological parameters.
   - What are “these climate conditions”? This sentence is confusing. Are you trying to say that temperature can alter other environments?

Reply: Done.

Moreover, air temperature is one of the most influential factors in the evapotranspiration phenomenon, which is vital for managing water resources and agricultural activities.
- “phenomenon” can be deleted
Reply: Done.
Furthermore, it has been observed that the temperature varies significantly, which may be responsible for the changes in weather throughout the time [41]
- This in unclear. Are you referring to seasonal variation of temperature or year-to-year variation of extreme temperatures?
Reply: its corrected.
The upper air patterns of each season have distinctive features, bringing several weather conditions.
- What are the distinctive features and several weather conditions? Elaborate or delete this sentence.

Reply: its deleted accordingly.

Accordingly, the continental climate of North Dakota makes the forecasting of the weather patterns a problematic task.
- Why is it difficult to forecast the weather continental climate compared to other climates? Please elaborate.

Reply: it was elaborated as suggested.

Tables 1 and 2 show the statistical characteristics of the minimum, mean, average, standard deviation, and skewness of the daily and weekly air temperature at the Cray meteorological station from 2000 to 2021.
- Is it Cray or Crary? I could not find station named Cray. Please make sure.
Reply: it is corrected. After checking it was Crary.

3. Are all the diagrams for the models (Figs 2-4) really necessary? I advise to delete these figures.
   Reply: they are removed as suggested.
   There are lots of interesting figures, but it does not get talked in the main text. Please give some elaboration of figures in the main text.

Reply: the figures have been explained in the text accordingly.

**References**

[3]	X.-D. Bai, W.-C. Cheng, and G. Li, “A comparative study of different machine learning algorithms in predicting EPB shield behaviour: a case study at the Xi’an metro, China,” *Acta Geotech.*, vol. 16, no. 12, pp. 4061–4080, 2021, doi: 10.1007/s11440-021-01383-7.

[4]	P. R. Cohen and D. Jensen, “Overfitting explained,” in *Sixth International Workshop on Artificial Intelligence and Statistics*, 1997, pp. 115–122.

[5]	X. Zhou, P. Lu, Z. Zheng, D. Tolliver, and A. Keramati, “Accident prediction accuracy assessment for highway-rail grade crossings using random forest algorithm compared with decision tree,” *Reliab. Eng. Syst. Saf.*, vol. 200, p. 106931, 2020.
