# Supplementary material for: Data-driven models for atmospheric air temperature forecasting at a continental climate region
Source: PLoS One. 2022 Nov 3;17(11):e0277079. doi: 10.1371/journal.pone.0277079 (PMC9632800; doi:10.1371/journal.pone.0277079)
Supplement: Attachment — Submitted filename: Response to Reviewers.docx [file pone.0277079.s002.docx]

Dear Editor,

Thank you for giving us the opportunity to submit a revised draft of our manuscript titled
"Data-Driven Models for Atmospheric Air Temperature Forecasting at a Continental Climate Region" to *PLOS One journal.* The authors appreciate the time and effort that you and the reviewers have dedicated to providing your valuable feedback on my manuscript. We are grateful to the reviewers for their insightful comments on our paper. Besides, we have been able to incorporate changes to reflect of the suggestions provided by the reviewers. Notably, the changes in the manuscript have been highlighted.

Here is a point-by-point response to the reviewers' comments and concerns.

**Comments from Reviewer**

**Reviewer #2: I appreciate the effort authors made to improve the paper. I think this is publishable after minor revisions**

1. This paragraph is unclear

North Dakota is located in the middle of North America and is subjected to extreme climate conditions, with hot summers and cold winters.

- Isn’t this the same for all regions? Why did you select North Dakota?

Reply:

- Yes, because they are located in a continental climate. One of the characteristics of this climate is that there is a difference between temperatures in summer and winter.
- The reasons of selecting the North Dakota are:

1- The location of the case study is in a climate where there is a significant change in the temperatures throughout the seasons.
2- Data availability.
3- It has a remarkable location. The distance from North Dakota to Equator and North Pole are almost the same.
4- All the above points were incorporated in the revised manuscript.

Furthermore, it has been observed that the temperature varies extremely from season to season, which may be responsible for the changes in weather throughout the time [42].

- Same. Of course, weather is causing temperature variation. Why is ND special? Or does it represent the general climate in continental US?

Reply: Due to its far inland location and proximity to both the North Pole and the Equator, which is almost equal, there are noticeable temperature fluctuations. We have incorporated this this point in the revised manuscript.

The continental climate of North Dakota makes forecasting weather patterns a problematic task. The difficulty in forecasting may be related to the climate throughout the year. Knowing that the high variation in temperatures through the seasons may hinder predicting the temperature

- It is confusing what the authors are trying to tell us. Please clarify.

Reply: Thank you for pointing out this problem. The authors with the best of their efforts have provided more clarity on this sentence as per the recommendations of the reviewer.

2. On SVR method, authors say: “14 However, this equation is unreliable in many hydrological challenges (nonlinear- regression analyses)”. However, the authors are predicting temperature, which is not hydrological (it might be in some aspect, but not generally). Please clarify or make it consistent.

Reply: The authors have now clarified this sentence as per the reviewers’ recommendations. The sentence is now more lucid and consistent for the readers.

3. This is clearly wrong: The first reason may be associated with the length of the data set used to develop the models. The St.D of the temperature records in February is very high (St.D = 7.695). The second significant reason is the data length used in this study. The first reason is associated with variability of temperature in wintertime, not the length of the data. Furthermore, the authors claim this is due to the insufficient data (in second reason). I assume this is because Feb has only 28 days, compared to other months with 30, 31 days. Do authors think 2-day (or about 8%) difference really make a huge difference in prediction performance? This can be tested by shortening other months to 28 days. If authors are going to claim this, it should be tested. However, I think this is not the main driver that causes the performance difference. The performance difference is also shown on weekly analysis, in which Feb has probably the same lengths (4-5 weeks) as other months.

Reply: The authors are very thankful to the reviewer for pointing out this issue. The authors totally agree with the point raised by the reviewer which says that the length of data is not the reason which makes a considerable difference in the prediction error. However, the variation of temperature may be the main reason and accordingly the authors have adjusted the manuscript based on the reviewers’ comments. Furthermore, the authors also have focused on the effects of extreme values on model performance.

4. The last reason may be related to the extreme negative values. This case study has a slight increase in temperature over time; therefore, there are more days with high temperatures than days with low temperatures. I don’t think trend in temperature really matters. Authors are claiming that there are very few extreme-low temperature to be trained, and this is mostly due to high variability of temperature in wintertime, which overlaps with the first reason.

Reply:

1. The authors completely agree with the reviewer that the first reason related to data duration does not have a significant effect in the context of model performance. Therefore, as per the reviewer’s suggestions, the authors have removed it from the entire manuscript and have disused this issue concerning the variability of data.
2. Moreover, the authors want to clarify that the negative extreme values interpret the performance of the model for specific months when temperatures are very low (i.e., January, February, and December). For these months, the negative extreme values are fewer in comparison to other months (see table 1) leading to higher forecasting error (see figure 7).

5. Same. Please make more high-level analysis on the reason: Moreover, the advanced analysis of forecasting error exhibits that the performance of the models is significantly affected by the length, consistency, and variability of data. As February has a fewer number of days as well as higher variability of recorded data, the monitored error in this month is higher than in other months.

Reply: More high-level analysis is performed accordingly. please see pg 27 , line number 4-9
